# Supplementary material for: Exploring Patient Empowerment in Major Depressive Disorder: Correlations of Trust, Active Role in Shared Decision-Making, and Symptomatology in a Sample of Italian Patients
Source: J Clin Med. 2024 Oct 21;13(20):6282. doi: 10.3390/jcm13206282 (PMC11508731; doi:10.3390/jcm13206282)
Supplement: Supplementary file 1 [file jcm-13-06282-s001.zip › jcm-3249244-supplementary.pdf]

Supplementary Table S1. Pharmacological interventions and nonpharmacological therapies of our sample.

**Antidepressants (dosage range)**	**N (%)**
Sertraline (50-100 mg/die)	9 (6.6)
Citalopram (20-40 mg/die)	13 (9.5)
Escitalopram (20 mg/die)	9 (6.6)
Fluoxetina (60 mg/die)	4 (2.9)
Paroxetina (10-20 mg/die)	6 (4.4)
Venlafaxine (75-225 mg/die)	8 (5.8)
Duloxetine (30-120 mg/die)	7 (5.1)
Trazodone (75-300 mg/die)	9 (6.6)
Vortioxetine (20 mg/die)	8 (5.8)
**Antipsychotics (dosage range)**	
Apripiprazole (5 mg/die)	3 (2.2)
Amisulpride (25 mg/die)	1 (0.7)
Olanzapine (5-10 mg/die)	5 (3.7)
Brexpiprazole (3 mg/die)	2 (1.5)
Quetiapine (50-100 mg/die)	4 (2.9)
Lurasidone (37mg/die)	1 (0.7)
Risperidone (1-2 mg/die)	4 (2.9)
**Stabilizers (dosage range)**	
Lithium sulfate (166 mg/die)	2 (1.5)
Lithium carbonate (150 mg/die)	1 (0.7)
Lamotrigine (50 mg/die)	4 (2.9)
Valproic acid/Sodium valproate (500 mg/die)	3 (2.2)
Sodium valproate (300 mg/die)	1 (0.7)
Pregabalin (50-100 mg/die)	6 (4.4)
**Benzodiazepines (dosage range)**	
Triazolam (250 mg/die)	9 (6.6)
Prazepam (10 mg/die)	2 (1.5)
Delorazepam (2 mg/die)	5 (3.7)
Estazolam (2 mg/die)	2 (1.5)
**Other treatments**	
Psychotheraphy	24 (17.5)

Supplementary Table S2. Sociodemographic variables and scale measures correlations (Kendall τ).

Variable	Scale	τ	p	95% CI Lower	95% CI Upper	Sig. Result	TOST Result
Duration of Illness	CDMS Total	-0.00476	0.954	-0.15936	0.15008	FALSE	TRUE
	SEMS Total	0.05256	0.525	-0.103	0.20561	FALSE	TRUE
	Tios Total	-0.14963	0.071	-0.29747	0.00521	FALSE	TRUE
	HAM-D Total	0.07735	0.349	-0.07831	0.22933	FALSE	TRUE
Education	CDMS Total	-0.07005	0.449	-0.22237	0.0856	FALSE	TRUE
	SEMS Total	-0.05464	0.554	-0.20761	0.10094	FALSE	TRUE
	Tios Total	0.16082	0.083	0.00625	0.30788	FALSE	FALSE
	HAM-D Total	0.09964	0.28	-0.05594	0.25051	FALSE	TRUE
Sex	CDMS Total	-0.04	0.684	-0.19353	0.11544	FALSE	TRUE
	SEMS Total	0.16447	0.094	0.00999	0.31127	FALSE	FALSE
	Tios Total	-0.10427	0.29	-0.25488	0.05129	FALSE	TRUE
	HAM-D Total	-0.1195	0.223	-0.26924	0.03589	FALSE	TRUE
Marital Status	CDMS Total	-0.06665	0.498	-0.21912	0.08899	FALSE	TRUE
	SEMS Total	0.14653	0.136	-0.00838	0.29458	FALSE	TRUE
	Tios Total	0.1138	0.248	-0.04166	0.26388	FALSE	TRUE
	HAM-D Total	0.03427	0.727	-0.1211	0.188	FALSE	TRUE
Living Condition	CDMS Total	-0.01346	0.891	-0.16784	0.14156	FALSE	TRUE
	SEMS Total	-0.104	0.29	-0.25463	0.05155	FALSE	TRUE
	Tios Total	-0.01954	0.843	-0.17374	0.13559	FALSE	TRUE
	HAM-D Total	0.01809	0.854	-0.13702	0.17234	FALSE	TRUE
Employment Status	CDMS Total	-0.20446	0.038	-0.34817	-0.05136	FALSE	FALSE
	SEMS Total	0.07581	0.440	-0.07985	0.22787	FALSE	TRUE
	Tios Total	-0.02030	0.837	-0.17448	0.13484	FALSE	TRUE
	HAM-D Total	0.01458	0.882	-0.14046	0.16893	FALSE	TRUE
